# Echinocandin Treatment of *Pneumocystis* Pneumonia in Rodent Models Depletes Cysts Leaving Trophic Burdens That Cannot Transmit the Infection

**DOI:** 10.1371/journal.pone.0008524

**Published:** 2010-01-29

**Authors:** Melanie T. Cushion, Michael J. Linke, Alan Ashbaugh, Tom Sesterhenn, Margaret S. Collins, Keeley Lynch, Ronald Brubaker, Peter D. Walzer

**Affiliations:** 1 College of Medicine, University of Cincinnati, Cincinnati, Ohio, United States of America; 2 Veterans Administration Medical Center, Cincinnati, Ohio, United States of America; 3 Department of Pathology, The Christ Hospital, Cincinnati, Ohio, United States of America; Albert Einstein College of Medicine, United States of America

## Abstract

Fungi in the genus *Pneumocystis* cause pneumonia (PCP) in hosts with debilitated immune systems and are emerging as co-morbidity factors associated with chronic diseases such as COPD. Limited therapeutic choices and poor understanding of the life cycle are a result of the inability of these fungi to grow outside the mammalian lung. Within the alveolar lumen, *Pneumocystis* spp., appear to have a bi-phasic life cycle consisting of an asexual phase characterized by binary fission of trophic forms and a sexual cycle resulting in formation of cysts, but the life cycle stage that transmits the infection is not known. The cysts, but not the trophic forms, express β -1,3-D-glucan synthetase and contain abundant β -1,3-D-glucan. Here we show that therapeutic and prophylactic treatment of PCP with echinocandins, compounds which inhibit the synthesis of β -1,3-D-glucan, depleted cysts in rodent models of PCP, while sparing the trophic forms which remained in significant numbers. Survival was enhanced in the echincandin treated mice, likely due to the decreased β -1,3-D-glucan content in the lungs of treated mice and rats which coincided with reductions of cyst numbers, and dramatic remodeling of organism morphology. Strong evidence for the cyst as the agent of transmission was provided by the failure of anidulafungin-treated mice to transmit the infection. We show for the first time that withdrawal of anidulafungin treatment with continued immunosuppression permitted the repopulation of cyst forms. Treatment of PCP with an echinocandin alone will not likely result in eradication of infection and cessation of echinocandin treatment while the patient remains immunosuppressed could result in relapse. Importantly, the echinocandins provide novel and powerful chemical tools to probe the still poorly understood bi-phasic life cycle of this genus of fungal pathogens.

## Introduction

*Pneumocystis* spp. are yeast-like fungi that reside extracellularly in lung alveoli and can cause a lethal pneumonia (PCP) in mammalian hosts with debilitated immune systems. Microscopic observations and molecular genetic studies suggest a life cycle that contains an asexual mode of replication via binary fission of the trophic form and a sexual mode resulting in formation of an ascus (cyst) containing 8 ascospores [1], [2]. Budding has not been observed in this genus. Mating is likely mediated by the trophic forms, as evidenced by several mating associated yeast homologs present in the *P. carinii* genome [3] and the expression of a pheromone receptor protein on the surface of the trophs [4]. The infection is thought to be initiated by attachment of the trophic forms to the Type I pneumocyte in the host alveoli. However, the mode of travel by the trophs to the alveoli is unknown, nor has the actual infectious propagule been identified. Transmission from one host to another is thought to be via an airborne route, but there is some evidence supporting alternative transmission through direct contact [5]. No environmental form or cycle has been identified.

Although the incidence of *P. jirovecii* Pneumonia (PCP) has decreased in industrialized countries with the introduction of Highly Active Anti-Retroviral Therapy (HAART) in 1996, PCP remains the leading opportunistic infection among HIV-infected patients and a serious clinical problem [6]. The mortality rate associated with PCP prior to and after the era of HAART has not changed significantly in the United States from an average of 10–15% [6], [7]. In developing countries and within urban areas of the United States, mortality is much higher, despite the availability of HAART [8]. The recent detection of *Pneumocystis jirovecii* in new patient populations such as those with chronic diseases states like chronic obstructive pulmonary disorder (COPD) [9], [10] or in those receiving anti-TNF therapy and other immunosuppressive agents [11], [12] demands a better understanding of the kinetics of its bi-phasic life cycle and the role each plays in establishment of infection, persistence, transmission, and pathology.

Standard antifungal drugs targeting ergosterol and ergosterol biosynthesis such as amphotericin B and the azoles, are ineffective against PCP [13]. The primary therapy for PCP is the combination of the anti-folate inhibitors trimethoprim-sulfamethoxazole (TMP-SMX) together with adjunctive corticosteorids. However, there are significant prophylactic breakthroughs and treatment failures associated with this combination, and second line therapies such as clindamycin-primaquine, atovaquone, or pentamidine have high rates of relapse and recurrence [14], [15]. Pentamidine and TMP-SMX both have significant side effects including nephrotoxicity and in the case of TMP-SMX, severe rash, fever, and neutropenia that often necessitate a change to alternative therapy. Long-term in vitro propagation of *Pneumocystis* spp. is not possible and the development of potential new therapies has necessarily been conducted using rodent models of infection. Because of the limited alternative anti-PCP therapies, physicians have resorted to using salvage therapies such as the echinocandins.

Echinocandins are a relatively new family of anti-fungal compounds that exhibit fungicidal activity against *Candida* species, including triazole-resistant isolates, and fungistatic activity against *Aspergillus* species [16]. Echinocandins inhibit synthesis of β -1,3-D-glucan, an essential component of the cell wall of many fungi, including the cyst form of *Pneumocystis* spp., not present in mammalian cells.

Reports of the efficacy of the echinocandins on PCP in patients have been contradictory [17], [18], [19], [20], [21]. This is due to the anecdotal nature of the reports, the low numbers of patients receiving treatment, and the use of the echinocandin as salvage therapy concomitant with other treatment. Except for studies performed in the 1990s which focused on caspofungin and FK463, (later called micafungin) [22], there have been no other studies of these compounds in animal models of *Pneumocystis* spp. [23], [24]. In the caspofungin studies (23), it was proposed that the cyst stage was required for ‘trophozoite” proliferation, but this was not tested directly. In addition, the question of whether the cysts or infection could return after stopping therapy was never addressed.

Because of the potential clinical use of the currently available echinocandins: anidulafungin, caspofungin and micafungin, and to address questions regarding effects on the life cycle and transmission of *Pneumocystis* spp., we conducted the first systematic evaluation of β -1,3-D-glucan synthase inhibitors in the mouse model of PCP with verification in the rat model. We tested the hypothesis that the cyst cycle was necessary for completion of the life cycle; whether it was the agent of transmission for *Pneumocystis* pneumonia; and if the echinocandin treatment would block repopulation of the cysts after treatment cessation. The consequences of these compounds on survival, organism morphology, and β-1,3-D-glucan content in the infected mouse lung were evaluated to provide a comprehensive picture of their myriad of effects on *Pneumocystis*.

## Results

### Therapy with the Echinocandins Targets the Cyst Form

**Figure 1:**
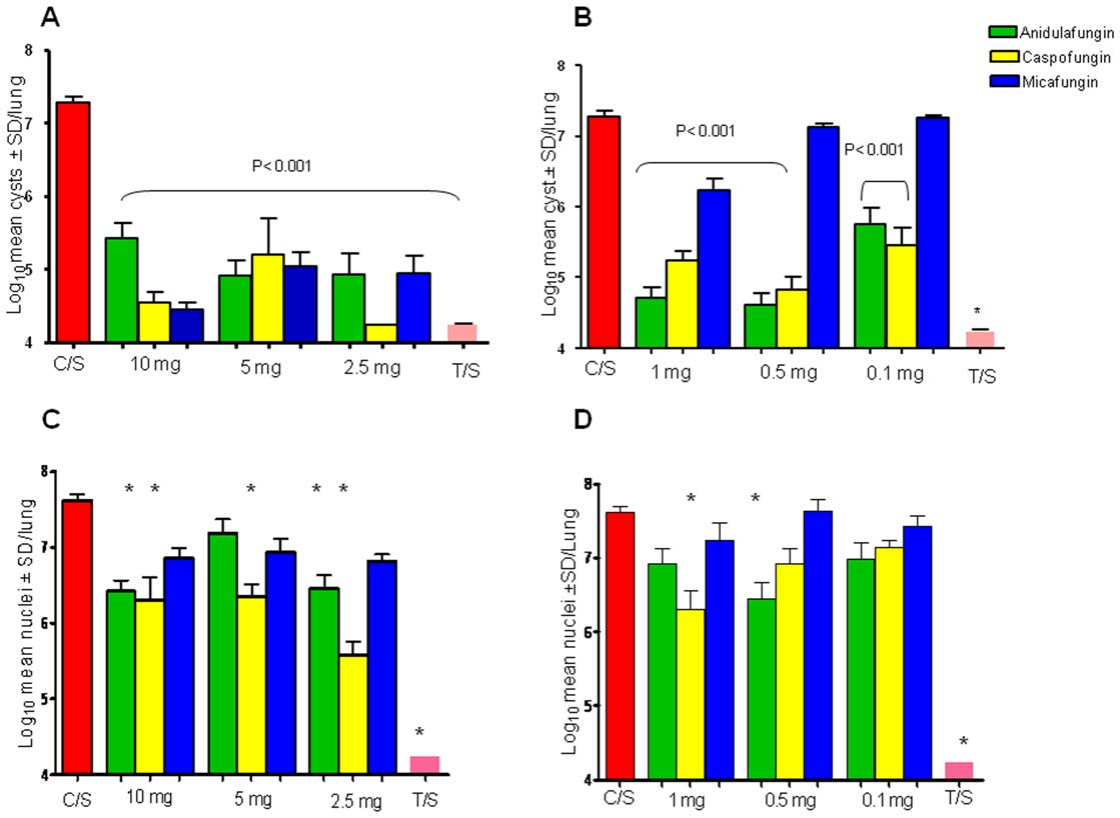
Therapeutic effects of anidulafungin, caspofungin and micafungin on *P. murina* cysts and trophic forms. Data in all panels represents mice treated 3 times per week for 3 weeks with anidulafungin (green bars), caspofungin (yellow bars), micafungin (blue bars) at the doses noted and trimethoprim-sulfamethoxazole at 50/200 mg/kg (pink bars, T/S) or untreated (red bars, C/S). Bars indicate log_10_ mean cysts±standard deviation (SD) per lung. Panel A: High dose echinocandin treatment, cyst burdens. All groups were significantly different than the untreated control mice (C/S), P<0.001. Panel B: Low dose echinocandin treatment, cyst burdens. Cyst burdens were compared to untreated controls (C/S) and P values for each group are listed on the graph; (*) indicates P<0.001. Panel C: High dose echinocandin treatment, trophic burdens *P<0.001 treated vs untreated control mice (C/S). Panel D: Low dose echinocandin treatment, trophic burdens. *P<0.001 treated vs untreated control mice.

Therapeutic treatment with anidulafungin, caspofungin and micafungin, administered 3 times per week for 3 weeks significantly reduced cyst burdens by 2 logs or more vs untreated mice (C/S; Control, Steroids) at doses of 10- to 2.5 mg/kg, (Figure 1a). With the exception of 10 mg/kg anidulafungin, all of the echinocandins at these dose levels were not significantly different than TMP-SMX (T/S), nor did they differ in efficacy from one another. At all of the lower doses (1.0- to 0.1 mg/kg), anidulafungin and caspofungin significantly reduced cyst burdens, while micafungin reduced cysts only at 1 mg/kg (Figure 1b). No dose response was observed for any echinocandin.

In contrast to the dramatic reductions of cysts, large numbers of trophic forms remained in the lungs of treated mice, often reaching levels that were not significantly different than untreated controls (Figure 1c,d.). Micafungin was the least effective with no significant reduction in trophic burden at any dose vs untreated control mice (C/S). The overall reductions were modest with a range from 1.1 log (10 mg/kg caspofungin) to 2 logs (2.5 mg/kg caspofungin). In contrast, TMP-SMX reduced the log_10_ *P. murina* nuclear count to undetectable levels. At the lower doses (Figure 1d), only caspofungin at 1 mg/kg and anidulafungin at 0.5 mg/kg were able to significantly reduce the trophic burdens as compared to the untreated mice. There was no dose response within any echinocandin series.

### Survival Is Enhanced by Echinocandin Treatment

**Figure 2:**
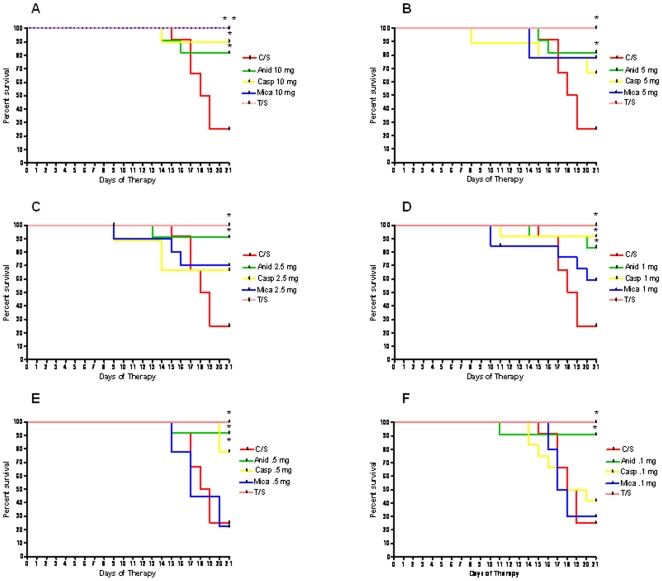
Survival curves of mice treated therapeutically with anidulafungin, caspofungin, micafungin. Percent survival for treated vs untreated mice was calculated for the 21 day treatment period using GraphPad Prism v.4. Asterisks indicate a significant difference from untreated controls (C/S). Line colors reflect the treatment and regimen as shown on the figure legend. Panel A: 10 mg/kg treatment regimen: anidulafungin: P = 0.02; caspofungin: P = 0.006; micafungin: P = 0.0006; T/S: 0.002. Panel B: 5 mg/kg treatment regimen: anidulafungin: P = 0.002; T/S: P = 0.02; Panel C: 2.5 mg/kg regimen: anidulafungin: P = 0.004; T/S: P = 0.002; Panel D: 1.0 mg/kg regimen: anidulafungin: P = 0.0049; caspofungin: P = 0.002; T/S: P = 0.002; Panel E: 0.5 mg/kg regimen: anidulafungin: P = 0.002; caspofungin:P = 0.007; T/S: P = 0.002; Panel F: 0.1 mg/kg regimen: anidulafungin: P = 0.004; T/S: P = 0.002.

Despite large trophic burdens, many of the treated mice had enhanced survival as compared to untreated mice (Figure 2). At the 10 mg/kg dose, all 3 echinocandins showed increased survival. Notably, anidulafungin treatment at each of the 6 doses provided significantly increased survival, while mice treated with caspofungin at doses of 1.0 mg/kg and 0.5 mg/kg only afforded increased survival rates. The survival rates were not associated with reductions in trophic burdens. For example, treatment with caspofungin at 2.5 mg/kg markedly reduced both cyst and trophic burdens, but did not increase survival as compared to untreated controls.

### Dramatic Morphological Remodeling

**Figure 3:**
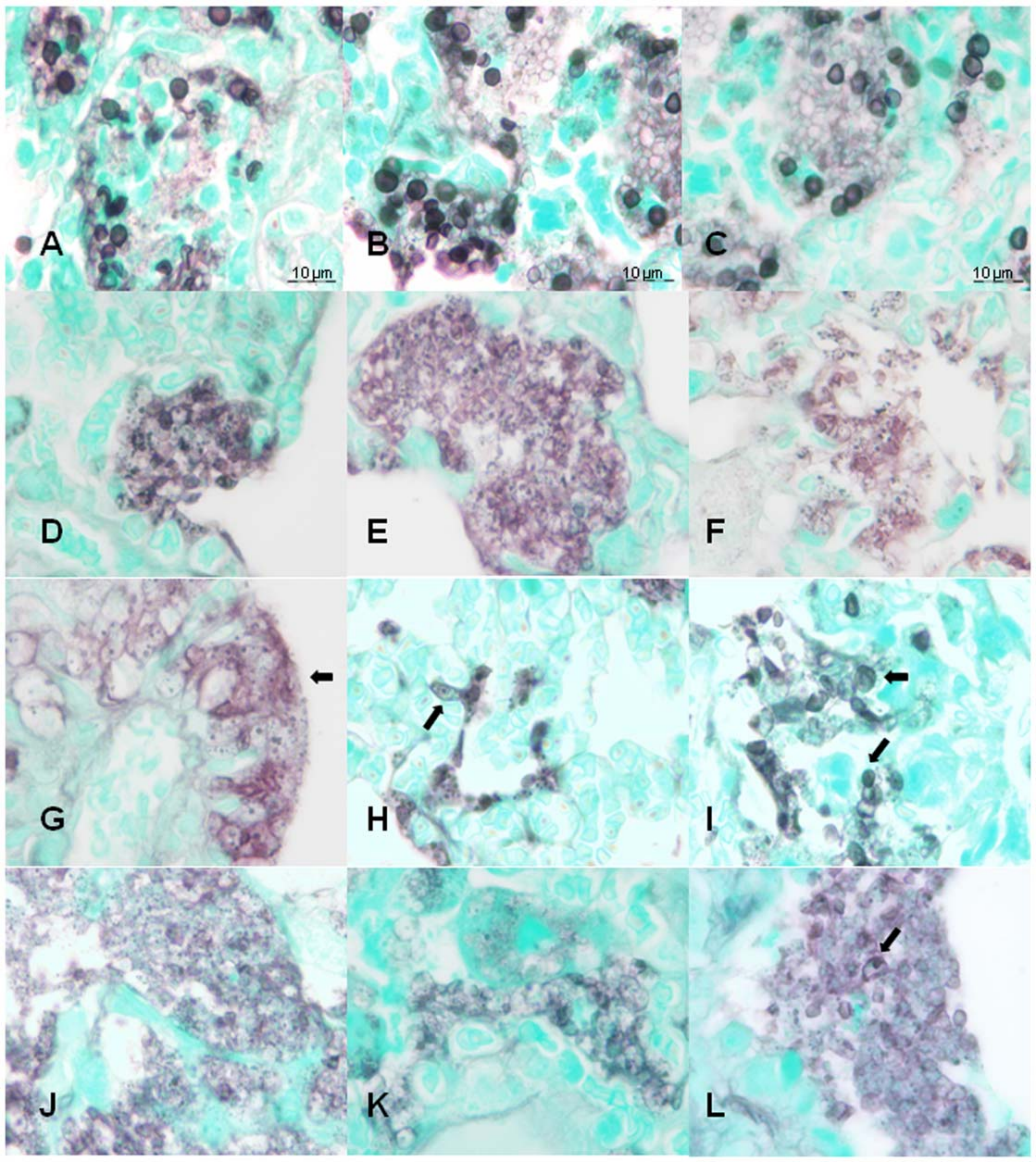
Histological morphology of *P. murina* treated with caspofungin, micafungin and anidulafungin. Grocott's methenamine silver-stained sections of lungs from: untreated mice (Panels A–C); representative sections from mice treated with 10-, 5- and 1 mg/kg caspofungin (Panels D–F;) representative sections from mice treated with 10-, 5- and 1 mg/kg micafungin (Panels G–I); and 10-, 5- and 1 mg/kg anidulafungin (Panels J–L). 1,250× magnification; 10 um bars in Panels A–C are the same for Panels D–L. Arrows indicate morphologies discussed in the text.

Similar to the effects on *Aspergillus fumigatus* [25], *P. murina* underwent profound morphological changes as a result of echinocandin treatment (Figure 3.). The silver impregnation of the *P. murina* in the lungs of the treated mice was noticeably diminished in the GMS (Grocott's methenamine silver) stained slides (Panels d-l) while those from the untreated mice exhibited strong silver staining and typical cup-shaped cyst morphology (Panels a–c). This was especially apparent in the mice treated with 10 mg/kg of any of the echinocandins (Panels d, g, j), where the organism infiltrates were characterized by diffuse staining without clearly circumscribed boundaries and the lack of cysts. Many of the organisms in 10- and 5 mg/ml dose treatments formed stacked coalescent structures on the alveolar epithelial cells suggestive of biofilms (See Panel g, for example). A prominent characteristic was the appearance of numerous GMS-staining bodies within the masses of the treated *P. murina.* These bodies showed great variation in size and shape and often appeared “fragmented” in a manner reminiscent of karyorrhexis in nucleated mammalian cells. Shown in Panel l (arrow) is an example of an enlarged cyst- like structure with just a single GMS-staining body. Distinct morphologies were observed in micafungin treated organisms. As shown in Panel h (5 mg/ml), *P. murina* from these mice often formed elongated structures reminiscent of abberrant hyphae. In mice treated with 1 mg/ml micafungin, cysts with typical morphologies were apparent (Panel i, arrow), which coincided with the reduced efficacy at this dose. Organisms from mice treated with caspofungin and anidulafungin, also exhibited unusual morphologies (Figure S1).

### Cysts Repopulate the Lungs after Cessation of Echinocandin Therapy

**Figure 4:**
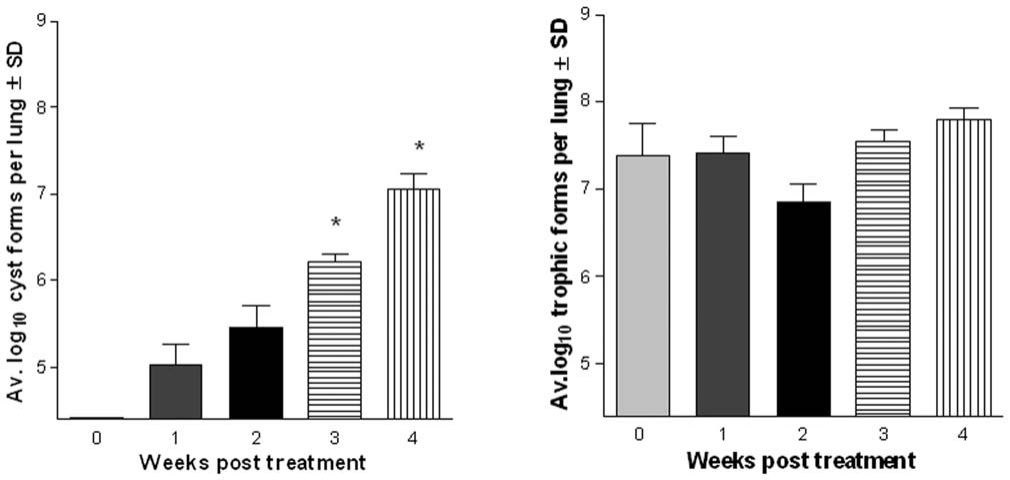
Cysts return after withdrawal of anidulafungin. (Panel A) Average log cyst counts. Week 2 vs 0 wk. (*) P<0.05; Weeks 4, 6 vs 0 wk (**) P<0.001 (Panel B) Average log trophic counts. No significant differences among the groups when compared to time point “0”. Weeks 2 and 4 post treatment were significantly different (P<0.05). The biological significance of this difference is unknown.

To address the question of whether cysts would repopulate the lungs of echinocandin- treated immunosuppressed mice after therapy was stopped, 2 studies were performed using only anidulafungin (1 mg/kg). No cyst forms were detected in the lungs of the mice that were examined immediately after stopping treatment, as expected (Figure 4a). Following withdrawal of the anidulafungin, cysts gradually repopulated the lungs over time (Figure 4a). After 2 weeks, significantly more cysts were detected compared to the 0 time point. By 4 weeks, cyst numbers reached the same level as untreated mice. Similar numbers of trophic forms were detected in all of the groups (Fig 4b).

### Verification of Cyst Targeting in the Rat Model of PCP

As in the mouse model, there was a marked decrease in cysts in the treated rats while trophic form numbers were similar to those from untreated rats. Untreated, steroid immunosuppressed rats had an average *P. carinii* log_10_ cyst count of 8.38±1.01 SD while the anidulafungin- treated rats had average cyst counts of 5.89±0.82 SD (P<0.01). The average *P. carinii* log_10_ trophs in the untreated, steroid immunosuppressed rats was 8.56±1.14 SD vs 7.38±0.69 SD in the anidulafungin treated group (P>0.05).

### Prophylaxis Resulted in Diminished Infections

**Figure 5:**
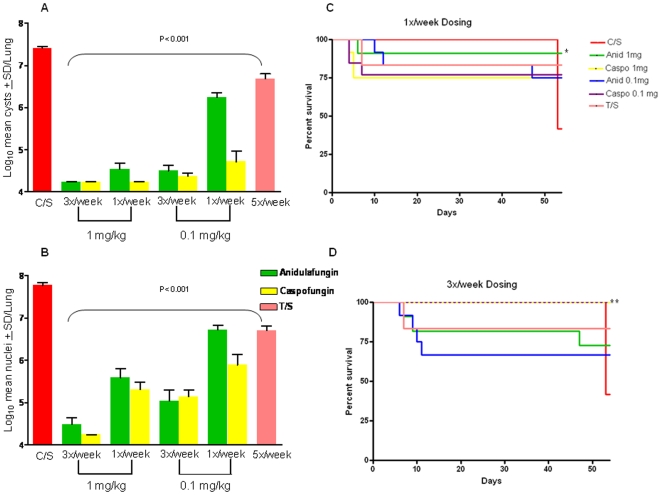
*Pneumocystis* pneumonia and survival curves of mice treated prophylactically with anidulafungin and caspofungin. Mice received prophylactic anidulafungin (green bars) and caspofungin (yellow bars) at doses at the dose regimens indicated on the figure legends. Trimethoprim-sulfamethoxazole (T/S) was given at the lower prophylactic dose of at 12.5/62.5 mg/kg. Bars indicate log_10_ mean cyst forms±SD/lung. Panel A: Log_10_ mean cyst counts of 1.0 and 0. 1 mg/kg caspofungin and anidulafungin. All treatment regimens were significantly different than untreated control mice (*P<0.001). Panel B: Log_10_ mean trophic counts of 1.0 and 0. 1 mg/kg caspofungin and anidulafungin. All treatment regimens were significantly different than untreated control mice (*P<0.001). Panel C: Survival curves of mice that received 1 mg/kg of anidulafungin and caspofungin once per week. 1X/week dosing: anidulafungin, 1 mg/kg: P = 0.03. Panel D: Survival curves of mice that received 1 mg/kg of anidulafungin and caspofungin thrice per week. Significance for 3X/week dosing: caspofungin, 1 mg/kg: P = 0.002; caspofungin, 0.1 mg/kg: P = 0.002.

Prophylactic treatment with low dose anidulafungin and caspofungin resulted in infections with diminished numbers of *P. murina*, but not complete inhibition. Treatment with either echinocandin at 1 mg/kg or 0.1 mg/kg given 3 times a week or once per week reduced cysts significantly when compared to the untreated controls (Figure 5a). Elevated cyst numbers were observed at 0.1 mg/kg administered only once per week for each compound, but even at these doses the cysts were significantly decreased vs untreated control mice. There were no statistical differences between the thrice weekly vs once weekly dosing at 1 mg/kg for either anidulafungin or caspofungin, but caspofungin and anidulafungin at 0.1 mg/kg, administered three times per week, were more efficacious in reducing cyst burdens than anidulafungin at the same dose given once per week (P<0.001). Caspofungin at 0.1 mg/kg once per week was also more effective than anidulafungin at the same dose schedule (P<0.001). At the lower concentration of TMP/SMX used for prophylaxis, cysts were significantly reduced vs. untreated control; however, at this low dosage, cyst burdens in mice treated with caspofungin and anidulafungin were significantly lower than those treated with TMP/SMX in all of the regimens (P<0.01).

As observed in the therapeutic studies, trophic forms in the prophylactically treated mice were more refractive to the echinocandins than cysts (Figure 5b), but mice treated with any of the prophylactic regimens had trophic burdens significantly lower than untreated mice. The ability of prophylactic administration to inhibit a robust infection suggests that blocking of β- 1,3-D-glucan synthesis interfered with establishment of a fulminate infection by inhibiting cyst formation in the recipient mice. Low doses of the echinocandins may have permitted some cysts to excyst in the treated mouse lungs and establish asexual, trophic replication but without the ability to enter the cyst cycle, were impeded in their ability to establish robust infection.

Survival in the prophylactically treated groups was generally enhanced when compared to the untreated mice. When administered 3 times per week all mice survived in both of the 1.0- and 0.1 mg/kg caspofungin groups as compared to 42% in the untreated group (Figure 5d). Although the other treatment groups had most of the mice remaining at the experimental end point (67–83%), these data did not reach statistical significance. When administered once per week, only the group that received 1.0 mg/kg anidulafungin had a statistically significant better survival rate than untreated mice (Figure 5c). However, as in the thrice weekly schedule, the majority (≥75%) of mice survived in all the treatment groups.

### The β-1,3-D-Glucan Content Was Decreased in Echinocandin-Treated Rodent Lungs

**Figure 6:**
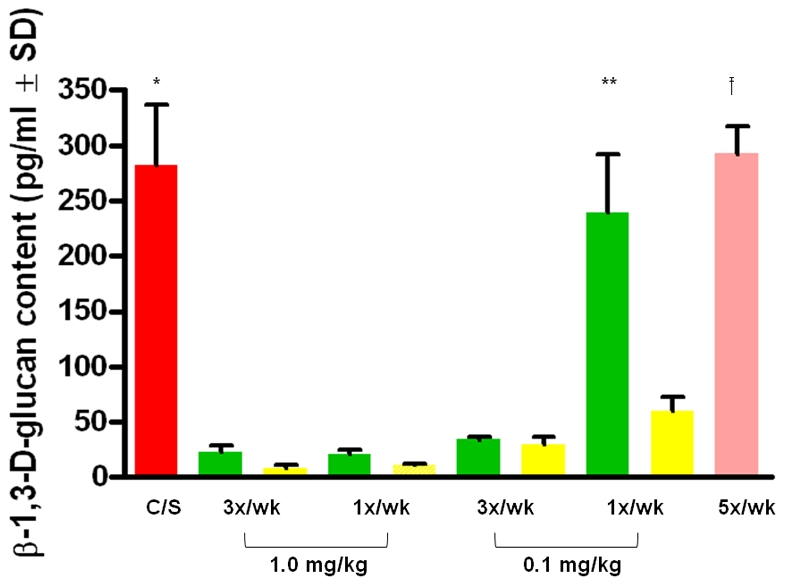
β-1,3-D-glucan in the lungs of mice treated prophylactically with echinocandins. β-1,3-D-glucan contents in the lungs of treated and untreated mice and rats were quantified with the GLUCATELL™ kit. Red bar (C/S) = untreated, immunosuppressed mice; Green bars = anidulafungin; Yellow bars = caspofungin; Pink bar = prophylactic dose of TMP/SMX (12.5/62.5 mg/kg). Dose regimens are described on the X-axis. (*) The average of the untreated mouse group was statistically different (P<0.001) from all groups treated prophylactically except anidulafungin 0.1 mg/kg/1x/wk and the TMP/SMX prophylactically -treated mice. (**)The average of the mice prophylactically treated with anidulafungin 0.1 mg/kg/1x/wk was statistically different from all of the echinocandin-treated mouse groups (P<0.001) but not from the TMP/SMX treated or the untreated mice. (^†^) The mice treated prophylactically with TMP/SMX treated were significantly different from all of the echinocandin-treated mice except for anidulafungin 0.1 mg/kg/1x/wk and the untreated control group (P<0.001).

The β-1,3-D-glucan content was measured in the lungs of the prophylactically treated mouse groups (shown in Fig. 5.) and the therapeutically treated rat groups (discussed above). As expected, the loss of cysts in the echinocandin-treated animals correlated with a reduction of β-1,3-D-glucan content (Fig. 6). The same 3 groups of mice that had elevated cyst numbers apparent in Fig. 5a also had dramatically higher β-1,3-D-glucan content (untreated control mice, mice treated with 0.1 mg/kg/1x/wk anidulafungin or low-dose TMP-SMX), shown in Fig. 6. In rats treated therapeutically with anidulafungin, the β-1,3-D-glucan content was significantly different from the untreated rat lungs, as were the cyst counts (857.4 pg/ml±611.3 SD in the untreated group and 90.6 pg/ml±134.3 S.D. in the anidulafungin treated group). These data show that the loss of cysts was proportional to the decrease in β-1,3-D-glucan content.

### Effects of Anidulafungin Exposure on Fluorescent Staining with a mAB Directed to β-1,3-D-Glucan

**Figure 7:**
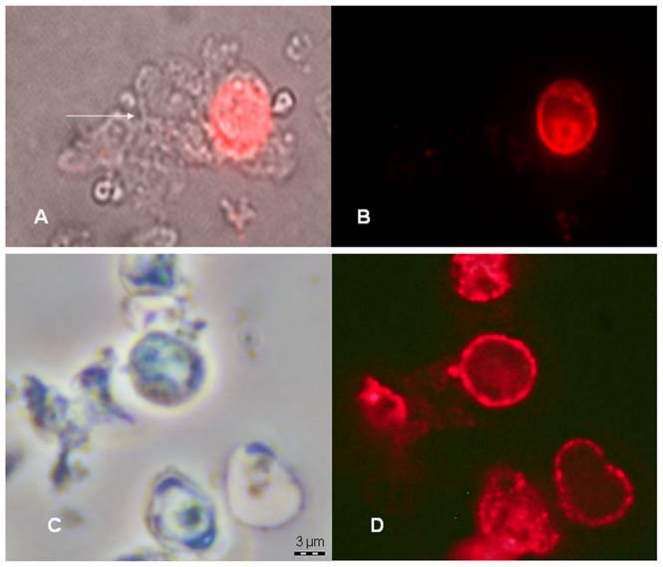
Effects of anidulafungin on β-1,3-D-glucan in *P. carinii*. *P. carinii* from the lungs of immunosuppressed rats were treated with 60 µg/ml anidulafungin for 24 hrs and reacted with a mAB to β-1,3-D-glucan conjugated to Alexafluor 594. Panel A: Non-anidulafungin treated *P. carinii* viewed by phase microsocopy showing a cluster of trophs (arrow) attached to a cyst; Panel B: the same cluster viewed by fluorescent microscopy; no trophs were stained by the antibody; Panel C: Anidulafungin-treated *P. carinii* viewed by phase contrast microscopy; and Panel D: the same cluster under fluorescent excitation. Note the punctate staining pattern in both samples. All panels magnified according to the bar in Panel C.

Fluorescent staining with a mAb specific for β-1,3-D-glucan was used to verify its presence in cysts and not in trophs; to evaluate the staining pattern of cysts; and to assess whether treatment with anidulafungin would lead to an “unmasking” of the β-1,3-D-glucan as reported for *C. albicans* [26], [27] and *A. fumigatus* [28], [29]. As expected, trophic forms did not stain while cysts emitted a robust fluorescence that appeared to be localized to the surface and in discrete round particles in untreated *P. carinii* (Figure 7a,b). Following treatment of *P. carinii* cysts with anidulafungin for 24 hours, no change in fluorescent intensity was apparent when evaluated by microscopic methods (Figure 7c,d) or by quantitiative measurement of fluorescent intensity (58,877 Fluorescent intensity units (FIU)±903 S.D. for the untreated control group vs. 47,834 FIU±5,647 for the anidulafungin-treated group, two-tailed P = 0.19).

### Evidence for the Cyst as the Agent of Transmission

**Figure 8:**
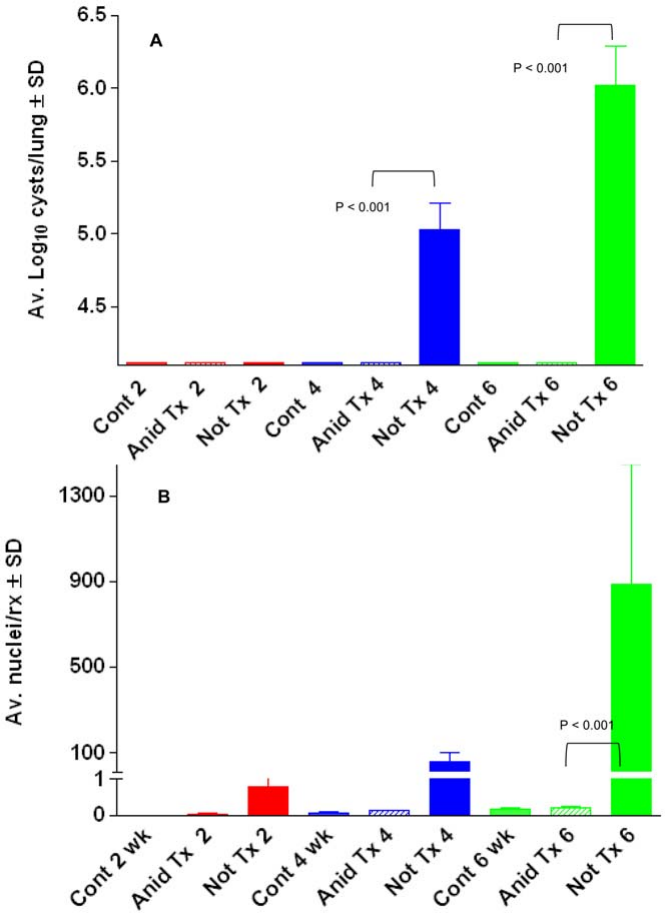
Anidulafungin-treated mice cannot transmit Pneumocystis infection. Panel A: Average cyst counts of mice exposed to untreated infected mice (Not treated, “Not Tx”) or anidulafungin treated (“Anid Tx”) mice and immunosuppressed for 2, 4 and 6 wks. “Controls” were non-exposed, immunosuppressed mice (“Cont”). Panel B: *P. murina* mtLSU message was detected by RT-qPCR and expressed as nuclei/reaction.

Anidulafungin was used as a chemical tool to interrogate the role of the cyst or trophic form as the agent of transmission. Results of these experiments demonstrated that anidulafungin treatment significantly reduced the ability of mice to transmit the infection by the seeding method, the most natural form of propagation of PCP (Figure 8a). No cyst forms were detected at any of the time points in recipient mice seeded with anidulafungin treated seed mice by microscopic evaluation (Anid Tx 2, 4, 6). *[Note log 4.24 is the level of sensitivity of microscopic evaluation; a value of 4.24 means no organisms were seen in 30 oil immersion fields]*. In recipient mice that were seeded with control, immunosuppressed but untreated mice (Not Tx 2, 4, 6), no cyst forms were detected after 2 wk time of immunosuppression; but log_10_ mean cyst counts per lung of approximately 5.0 and 6.0 were detected in the 4 and 6 wk groups (Fig. 8a, blue, green bars). No cyst forms were detected in any of the non-exposed immunosuppressed control mice (Cont 2, 4, 6).

To determine whether the anidulafungin treated mice possibly transmitted the infection at low levels, (below the limit of microscopic detection) the recipient mice were evaluated for the presence of *P. murina* by RT-qPCR analysis. The results of this analysis confirmed the findings of the microscopic evaluation (Fig. 8b.). Either no or very low levels (<1 *P. murina* nuclei/rx) of *P. murina* mtLSU message was detected by RT-qPCR analysis in the lungs of recipient mice that were seeded with mice that were treated with anidulafungin (“Anid”) at 2, 4, and 6 weeks post exposure. In mice that were seeded with the non-treated and infected control mice, mtLSU message was detected in all mice at all 3 time points in the lungs of exposed mice (Fig. 8b, Not Tx 2-, 4- and 6). At the 6 week time point, significantly higher levels mtLSU were detected in the mice seeded with untreated infected mice compared to mice seeded with treated mice (P<0.01). No mtLSU message was detected in any of the unexposed immunosuppressed control mice (Cont 2, 4, 6).

### Trophic Forms Remaining after Anidulafungin Treatment Are Viable and Infective

To evaluate whether the trophic populations isolated from anidulafungin treated mice were viable and infective, *P. murina* isolated from either the treated or non-treated control seed mice, were used to infect *P. murina-*naïve immunosuppressed mice by intranasal instillation of 10^7^nuclei. The mice were sacrificed at 2, 4, and 6 weeks post inoculation and evaluated for *P. murina* infection by RT-qPCR and microscopic enumeration, as described above.

**Figure 9:**
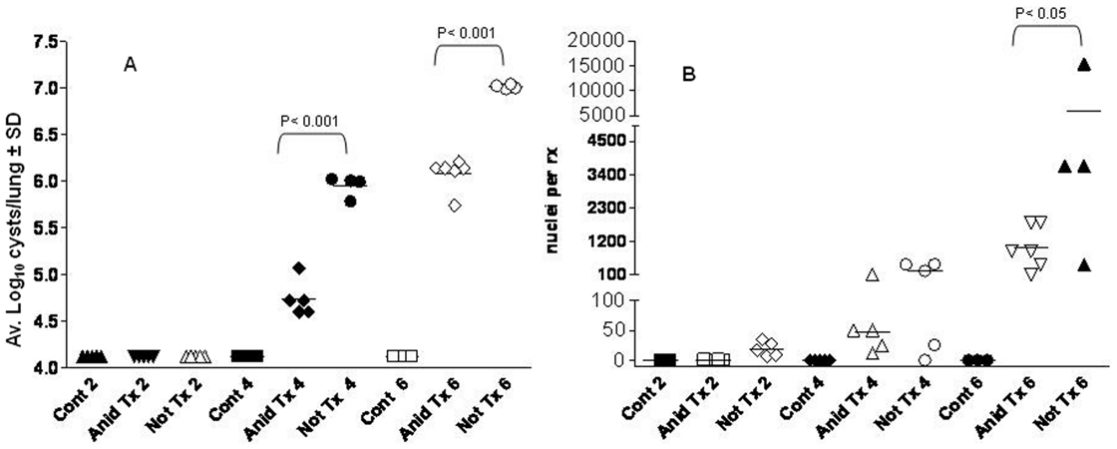
Intranasal inoculation of *P. murina* from anidulafungin treated mice cause infection and are viable. Panel A: Average log_10_ cysts in the lungs of immunosuppressed mice intranasally inoculated with anidulafungin treated or non-treated organisms. Not Tx = non-treated; Anid Tx = anidulafungin treated *P. murina*. 2-, 4-, 6 wks immunosuppression; Cont = immunosuppressed uninoculated mice. Panel B: RT-qPCR of immunosuppressed mice inoculated with anidulafungin-treated or non-treated *P. murina*. Legend as in Panel A. Significant differences between groups are shown on the figure.

No cysts were detected microscopically at 2 wks in mice that were inoculated with control or treated *P. murina* (Fig. 9a, Anid Tx 2, Not Tx 2). At 4 and 6 wks, log_10_ mean cyst counts of approximately 4.7 and 6.0 were detected in the groups of mice infected with anidulafungin-treated *P. murina* (Fig. 9a, Anid Tx 4 and Anid Tx 6). However, significantly higher numbers of cyst forms were detected in mice that were infected with *P. murina* from non-treated mice at these same time points (Not Tx 4, 6) (P<0.05). These mice had log mean cyst counts of approximately 6.0 and 7.0 per group at the same time points (Fig. 9a). No cyst forms were detected in control mice that were immunosuppressed and uninoculated (Cont 2,4,6).

By Reverse Transcriptase qPCR analysis (which also served as a viability assessment), an average of approximately 1, 48, and 1000 nuclei per rx (reaction) were detected in groups of mice at the 2, 4, and 6 wk time points in mice infected with treated *P. murina* (Fig. 9b, Anid Tx 2, Anid Tx 4, Anid Tx 6). In mice that received non-treated *P. murina*, an average of approximately 20, 200 and 6000 nuclei/rx were detected per group at the same time points (Fig. 9b, Not Tx 2, Not Tx 4, Not Tx 6). No mtLSU message was detected in any of the unexposed immunosuppressed control mice (Cont 2, 4, 6).

## Discussion

There are both significant clinical and biological implications of these studies. In regard to clinical significance, the results presented here show for the first time that there is a hierarchy of efficacy against PCP among the echinocandins. Caspofungin and anidulafungin were significantly better at reducing cyst burdens than micafungin. However, unlike treatment of *C. albicans* infections with caspofungin which results in a candicidal effect, treatment of *Pneumocystis* spp. with these compounds targeted the cysts, sparing the trophic forms and was not pneumocidal. This selectivity is more similar to the effects of echinocandins on *Aspergillus fumigatus*, which do not fully inhibit its growth and target the hyphal stage [25].

Our results strongly suggest that micafungin would not be a suitable choice for clinical use and any echinocandin treatment should not be administered as a mono-therapy, as the infection is not eradicated. The withdrawal of anidulafungin treatment and subsequent cyst repopulation also pose a cautionary note for the clinical use of echinocandins to treat *Pneumocystis jirovecii* pneumonia, as cessation of therapy could result in relapse. However, administration of an echinocandin in combination with TMP-SMX (or other therapy) could provide significant benefits by decreasing the inflammatory responses associated with β-1,3-glucan and hastening the clearance of the infection, as they are faster acting than TMP-SMX. It is possible these effects would lead to lower mortality rates as well. Administration of echinocandins may be particularly useful during immune reconstitution syndrome as a replacement for corticosteroids, which are recommended within 72 hours of diagnosis in cases of moderately or severe PCP [30], since the echinocandins do not have the pan-immunosuppressive effects of corticosteroids and are virtually non-toxic to mammals.

Like results reported for *Aspergillus fumigatus* [25], *P. murina* underwent profound morphological changes as a result of echinocandin treatment. The organisms appeared to coalesce into larger masses with fewer circumscribed boundaries and larger non-staining bodies. In some cases, the stacked organisms that lined the alveoli resembled biofilm structures previously observed in an in vitro Pneumocystis biofilm system [31]. We speculate that the trophic forms reside in these masses which afford some protection from the host immune system without activating it.

The echinocandins exhibited a bias of effect on the cyst stage of *P. murina* and *P. carinii* in vivo, which was likely due to the lack of β-glucan in the trophic stage. Similarly, treatment of *C. albicans* in vitro and in vivo with caspofungin resulted in a dramatic bias towards the filamentous morphotype while in *A. fumigatus*, the hyphae, but not the germlings or conidia were targeted. Concomitant with these selective effects was an “unmasking” or exposing of β-glucan that is naturally cloaked beneath a layer of mannan [26], [27], [28], [29]. Such unmasking events led to increased Dectin-1 mediated inflammatory responses to *A. fumigatus* hyphae [29] and *C. albicans* [32]. Unlike *C. albicans* or *A. fumigatus*, the fluorescent staining patterns or intensities of *P. carinii* were not effected by anidulafungin, which is in keeping with previous studies that showed the direct interaction of cysts with alveolar macrophages via this Dectin-1, without the need for prior treatment [33].

Another intriguing finding of these studies was the ability to prevent moderate to severe infection in mice prophylactically treated with low doses of caspofungin and anidulafungin, given as infrequently as once per week. These mice had dramatically reduced cysts and significantly lowered numbers of trophic forms when compared to untreated controls. Recipient mice treated with the echinocandins received the same exposure to infected mice as did the non-echinocandin treated mice who developed robust infections. This suggests that blocking of β-1,3-D-glucan synthesis interfered with establishment of a fulminate infection perhaps by inhibiting cyst formation in the recipient mice. These results provide some evidence that the cyst is the infectious agent that transmits the infection and also may be required for efficient replication in the mammalian hosts. Although the echincandins have the disadvantage of parenteral administration, the once weekly regimen offers an alternative to patients that cannot tolerate standard prophylaxis.

Studies exploiting the ability of anidulafungin treatment to provide mice with infections comprised almost entirely, if not entirely of trophic forms provided strong evidence that cysts are required for transmission, as these mice could not transmit the infection to immunosuppressed, uninfected mice via the natural, airborne route. Yet the trophic forms from the treated mice were viable and caused infection when directly inoculated into immunosuppressed mouse lungs, although these infections were generally lower in organism burden than their counterparts. This decrease in burden may have been due to unknown effects by the echincandin on trophic viability or due to a longer time period required to reach higher infection levels without cysts in the initial population. Nonetheless, these studies provide compelling evidence that the cyst form of *Pneumocystis* is required for natural, airborne transmission. In vivo, the cyst is 5–8 µm which is a sufficient diameter to permit deposition in the deep airways [34]. In addition, desiccation is known to reduce the size of fungal spores [35] and desiccation of the cysts by contact with air during the transmission route would further reduce the size resulting in even smaller aerosols that could remain airborne longer, increasing the chances of transmission. The added advantage of 8 ascospores contained within the cyst would potentially provide a more rapid establishment of infection and perhaps contain the mating types necessary to initiate sexual replication.

The lack of mortality associated with echinocandin treated mice may have been due to the lack of glucan, which has been shown to be a pro-inflammatory factor, with detrimental as well as beneficial immune responses [36], [37], [38], [39]. Previous studies have implicated β -1,3-D-glucan as a cause of a destructive inflammatory response that contributes to the disease process in *Pneumocystis* spp. pneumonia, however, these studies relied on responses from isolated alveolar epithelial cells, macrophages, or T-cells [37], [38], [40], isolated dendritic cells [39] or by installation of a glucan-enriched preparation from *P. carinii* into healthy mice [40]. Our studies extend and enhance the previous studies by showing that *in vivo* removal of the specific β -1,3-D-glucan component of the cyst using echinocandin treatment, reduced the mortality of the treated animals, even with significant trophic burdens remaining. The model can now be used to directly investigate immune responses to the individual stages both in vitro and in vivo.

Anidulafungin and caspofungin were able to modulate and regulate the *Pneumocystis* spp. life cycle. Inhibition of β-1,3-glucan synthesis lead to an infection that was comprised primarily, if not all, of trophic forms, which is presumed to be the asexual phase of the life cycle. Using these compounds, identification of the genes necessary for asexual vs sexual replication and those involved in the switch between the 2 phases can now be identified.

## Materials and Methods

All animals were handled in strict accordance with good animal practice as defined by the University of Cincinnati and VAMC IACUC and all animal work was approved by the appropriate committees: UC 06-03-23-01 and VA 06-06-02-01. The animal husbandry and experimental procedures are consistent with the recommendations in the Guide for the Care and Use of Laboratory Animals (the *Guide*), the Animal Welfare Act Regulations (AWARs), and the Public Health Service Policy on Humane Care and Use of Laboratory Animals (PHS Policy). To safeguard against environmental exposure of *P. murina* and other microbes, mice are housed under barrier conditions with autoclaved food, acidified water, and bedding in sterilized shoebox cages equipped with sterile microfilter lids [41]. Access is limited to animal care and technical personnel who are required to wear sterile caps, gowns, masks, gloves and booties while in the animal rooms. The animals are observed daily and those that appear gravely ill or moribund are routinely euthanized so as not to cause any undue suffering. Animals are euthanized by an overdose of 70% carbon dioxide in a precharged airtight chamber followed by bilateral pneumothorax, which is an approved euthanasia method according to the latest report of the AVMA Panel on Euthanasia.

### Compounds

Caspofungin (Cancidas–Merck Pharmaceuticals, Rahway, NJ), Micafungin (Mycamine–Astellas Pharma US), and Anidulafungin (Eraxis–Pfizer, New York, NY) were all purchased commercially as a powder. Trimethoprim-sulfamethoxazole (Sulfatrim®, Actavis, Baltimore, MD) was purchased as a pediatric oral suspension.

### *In vivo* Mouse Studies

#### Therapy

Evaluation of anti-*Pneumocystis* spp. activities was conducted with an immunosuppressed mouse model of pneumocystosis infected with *P. murina* as previously described [42]. C3H/HeN mice were exposed to previously infected mice for 2 weeks (a process termed, “seeding”, which is the most “natural” method of infection) and begun on an immunosuppressive regimen the first day of exposure (4 ug/ml dexamethasone in drinking water). Mice with moderate *P. murina* infections (4–5 weeks post exposure) were randomly divided into treatment and control groups of 12 each and begun on the treatment regimen or vehicle sham (C/S; Control, Steroids). The echinocandins were dissolved in sterile 0.9% sodium chloride and were administered by intraperitoneal (ip) injection on a mg/kg basis once daily for 3 days/week at varying dose levels and once per week for selected compounds. Trimethoprim-sulfamethoxazole (T/S) pediatric suspension was administered by oral gavage at 50 mg–250 mg per dose. The drug treatment continued for 3 weeks during which time the mice remained on the immunosuppressive regimen. Mice were examined daily for any signs of overt toxicity during the treatment phase as previously described [42].

#### Prophylaxis

The prophylaxis studies were performed in the same manner as described above except the mice were treated for the entire duration of the 7–8 week study with dexamethasone and echinocandin and exposed to *P. murina*-infected mice during the first 2 weeks of immunosuppression. A reduced regimen of 12.5/62.5 mgTMP-SMX was used as the prophylactic dose, based on previous studies in rats [43].

#### Evaluation of efficacy

Efficacy was based on a reduction in organism burden in treated vs untreated mice determined by microscopic quantification. The right lung was homogenized and slides were stained with cresyl echt violet (CEV), which selectively stains the cyst form of *P. murina* and a rapid version of the Wright-Giemsa stain (Leuko-stat, Fisher Scientific) to enumerate trophic forms [42]. The microscopic counts were log transformed and the group outcomes were compared by the one way ANOVA followed by Newman-Keuls multiple comparison post test using INSTAT v.4 (GraphPad software for Science, San Diego, CA). Significance was accepted when the P value was <0.05. The limit of detection by this enumeration method is 1.75×10^4^ (log_10_ 4.24/lung). Survival curves were based on the 21-day treatment period and were compared using GraphPad Prism v.4. Significance was accepted when the P value was <0.05.

#### Histological evaluation

The left lungs of 5 of the mice therapeutically treated with 10-, 5- and 1 mg/kg of the 3 echinocandins in each group and 3 from untreated controls were inflated with a solution of 10% buffered formalin solution and submitted to the University of Cincinnati Comparative Pathology Core for further processing. The sections were stained with Grocott's methenamine silver (GMS) which stains fungal cell walls including those of the cysts of Pneumocystis. All slides were examined in a blinded manner by a pathologist (RB). Morphological analyses were conducted using an Olympus BH2 microscope and an Olympus DP72 digital camera and software system.

### Withdrawal of Anidulafungin to Evaluate Return of Cyst Populations

Two separate studies were conducted using C3H/HeN and scid/scid mice. *P. murina* infections were induced in mice as described above for the therapeutic studies. Mice were then treated with 1 mg/kg anidulafungin for three weeks after which the treatment was stopped, but the immunosuppression was continued. Control mice received no treatment, but the same length of immunosuppression. Groups of 3–6 mice per group were sacrificed immediately after stopping the treatment and then at 1, 2, 3, and 4 weeks post cessation. The lungs were processed and examined microscopically for trophic and cyst forms as described above. The results from the 2 experiments were averaged and analyzed using descriptive statistics.

### Transmission Studies

To assess the ability of anidulafungin treated *P. murina* infected mice to transmit the infection, *P. murina-* naïve immunosuppressed C3H/HeN mice were co-housed (seeded) with anidulafungin- treated or control, untreated *P. murina-* infected C3H/HeN mice. The mice serving as the treated seed mice received anidulafungin at a dose 1 mg/kg, i.p. 3x per week. This treatment regimen results in elimination of almost all cyst forms from the lungs of the treated mice, but similar levels of trophic burdens remain in the untreated and anidulafungin treated mice (See results). Following the 3 weeks of anidulafungin treatment, the treated mice were then used as seed mice and the anidulafungin dosing was continued during the seeding period of 2 weeks.

One treated or untreated control mouse was co-housed in the same cage with 4–6 naïve immunosuppressed mice. To ensure that all recipient mice were exposed to a similar level of *P. murina* during the seeding, the seed mice were rotated between cages every 2–3 days to account for potential variations in *P. murina* infection levels between the seed mice. Following the 2 weeks of seeding, the control or treated seed mice were removed from the cages. The seed mice were sacrificed, and the lungs processed to evaluate the level of *P. murina* infection and for preparation of inocula as previously described [42].

The recipient mice were continued on immunosuppression and sacrificed at 2, 4, and 6 wks post initial exposure. Immunosuppressed mice with no experimental exposure to *P. murina* were also sacrificed at each time point, as controls.

#### Quantification by Reverse Transcriptase quantitative PCR (RT-qPCR)

To detect low level infections and to ensure that the organisms are viable, RT-qPCR was performed and compared to microscopic counts. At the time of sacrifice the lungs were flash frozen in liquid nitrogen, ground into a fine powder, extracted by Triazol®, quantified and stored at −70°C for subsequent microscopic (cyst enumeration) and quantitation of the *P. murina* large subunit ribosomal RNA gene (mtLSU) message levels [44]. The amount of *P. murina* large subunit ribosomal RNA gene (mtLSU) message was quantified using a previously described TaqMan assay [45]. The threshold cycle for each sample was identified as the point at which the fluorescence generated by degradation of the TaqMan probe increased significantly above the baseline. To convert the threshold cycle data to *P. murina* nuclei, a standard curve was generated using cDNA made from RNA isolated from 10^7^ *P. murina* nuclei. The level of infection of the samples was estimated using the standard curve. The efficiency of the standard curve PCR reactions consistently approached 100% in our hands. Importantly, detection of *P. murina* mtLSU with this assay has also been shown to correlate with viability of the organisms [33].

To ensure that high quality RNA was isolated from all samples and that cDNA synthesis was successful, a SybrGreen incorporation RT-PCR assay for the mouse vimentin gene mRNA was performed on all samples. Primers amplify a 109 base pair product from mouse vimentin mRNA. The fluorescent signal generated by incorporation of SybrGreen into the double-stranded product was collected at 86°C for 10 sec during each repeat to determine the threshold cycle for each sample. The fidelity of the PCR reactions was confirmed by analysis of the melt curve of the vimentin PCR product. A single peak with an approximate melting temperature of 88°C was detected as expected. The efficiency of the vimentin PCR reactions consistently approached 100% in our hands.

#### Verification of the viability of the anidulafungin-treated trophic forms

To evaluate whether the trophic populations isolated from anidulafungin treated mice were viable and infective, *P. murina* isolated from either the treated or non-treated control seed mice, were used to infect *P. murina-*naïve immunosuppressed mice by intranasal instillation of 10^7^nuclei in 50 ul as previously described [46]. The mice were sacrificed at 2, 4, and 6 weeks post inoculation and evaluated for *P. murina* infection by RT-qPCR and microscopic enumeration, as described above.

### *In vivo* Rat Studies

A small pilot study was conducted to ensure that the results in echinocandin- treated rats were similar to those in mice. 12 8 wk old CD rats (Charles River, Inc.) were received from the vendor, and their oral cavities swabbed to detect *P. carinii* colonization [47]. All rats were negative for the presence of *P. carinii.* After 1 week of corticosteroid treatment (20 mg/kg Depo-Medrol/wk)(Pfizer, New York, NY) all rats were intranasally inoculated with 2×10^7^ *P. carinii* nuclei in 0.1 ml of PBS. Rats were continued on immunosuppression for 5 weeks after which 6 rats were administered 5 mg/kg anidulafungin (Eraxis), for an additional 3 weeks while on steroids, while the other 6 received no treatment, and remained on the suppressive regimen. After the 3 wks, the rats were sacrificed and cyst and trophic forms were enumerated microscopically as described for mice.

### Measurement of β-1,3-D-Glucan in the Lungs

The β-1,3-D-glucan levels in echinocandin treated and untreated rodent lungs were measured in the mice from the prophylaxis study and rats from the therapeutic study (described above). The same homogenates used for enumeration of β-1,3-D-glucan-mAb and CEV-stained cysts were used for measurement of total β-1,3-D-glucan content. The GLUCATELL™ (Associates of Cape Cod, Inc.,East Falmouth, MA) endpoint assay was used according to the vendor's instructions. Aliquots were resuspended in 1 ml of 1 M sodium hydroxide and shaken at room temperature for 1 hour. Samples were prepared as 10-fold dilutions in pyrogen free water in test tubes certified to be free of glucan (Associates of Cape Cod). Glucan standards were prepared in the same manner according to vendor instructions, The (1,3)-ß-D-glucan content was measured by using a linear regression curve of standards from 25–200 pg/ml. The correlation coefficient for the studies presented was 0.992, within the acceptable range suggested by the vendor (>0.980). Glucan content was expressed as pg/ml. One tail P-value was calculated for the rat studies while ANOVA was used in the mouse study (GraphPad Instat v.3).

### Effects of Anidulafungin Exposure on Fluorescent Staining with a Monoclonal Antibody (mAb) Directed to β-1,3-D-Glucan

Cryopreserved, untreated, *P. carinii* were rapidly thawed in a 37°C water bath, washed with PBS, centrifuged at 1000×g, and suspended in RPMI 1640 supplemented with 5% calf serum with (treated) or without (control) 60 µg/ml anidulafungin at 10^8^ *P. carinii*/ml [48]. Each suspension was distributed in wells (3 ml) of a Corning 6-well plate (Corning NY) and incubated for 24 hr at 37°C, 5% CO_2._ Wells were harvested from control and treated wells, transferred to microfuge tubes, centrifuged at 1000×g for 2 min, and resuspended in 500 µl PBS with 1%BSA. 50 µl was removed for the ATP assay and 2.5 µl of 1.0 mg/ml β-1,3-D-glucan mAb (Biosupplies Australia, Victoria, Australia) was added and incubated at 37°C for 1 hr. The mAb does not cross react with 1,4-α-glucan nor (1→3, 1→4)-β-glucan. The preparations were centrifuged, washed and resuspended in the PBS solution to which 2.5 µl of Alexafluor 594 conjugate (InVitrogen, Molecular Probes, Inc.) was added. After incubation for 1 hr at 37°C, the preparations were washed and resuspended as above and examined under epifluorescence at excitation of 590 nm and emission of 617 nm, then photographed using an Olympus DP72-BSW camera and software system. The use of unfixed, live organisms is sometimes referred to as “ex vivo” fluorescence (evf) and is used because it preserves the live cell wall organization as opposed to cross-linking and other effects induced by fixation [27]. A series of 1∶2 dilutions of the washed fluorescent control and treated *P. carini* solutions were prepared in PBS. Aliquots of 100 µl of the series made from control and treated organisms were distributed in an opaque black 96-well plate (Corning, NY) and quantified for fluorescent intensity using a FluoroStar Optima plate reader (BMG Labtechnologies, Durham, NC) with an excitation filter of 590 nm and emission of 617 nm. Fluorescent intesity was determined by 20 flashes per well.

## Supporting Information

Figure S1 Unusual morphologies of *P. murina* in the lungs of mice treated with echinocandins. Grocott's methenamine silver (GMS)-stained sections of lungs from mice treated with anidulafungin and micafungin: Panel a: 10× magnification of ball-like structure in a mouse treated with 5 mg/kg anidulafungin. Note the clearly circumscribed boundaries of this ∼70 um structure. Panel b: Higher magnification of the structure. Panel c: Extension containing GMS-stained organisms in a mouse treated with 5 mg/kg micafungin. Panel d: Higher magnification of the tip of the elongation (1250X). Panel e: Chains of GMS-staining organisms resembling pseudohyphae in a mouse treated with 5 mg/kg micafungin. Panel f: Higher magnification of a different chain in the same mouse treated with 5 mg/kg micafungin. Panel g: An unusual elongated structure in a mouse treated with 5 mg/kg micafungin. Magnification bars are included for each micrograph.
(2.26 MB TIF)
