# Supplementary figures and images for: Echinocandin Treatment of Pneumocystis Pneumonia in Rodent Models Depletes Cysts Leaving Trophic Burdens That Cannot Transmit the Infection
Source: PLoS One. 2010 Jan 29;5(1):e8524. doi: 10.1371/journal.pone.0008524 (PMC2813285; doi:10.1371/journal.pone.0008524)

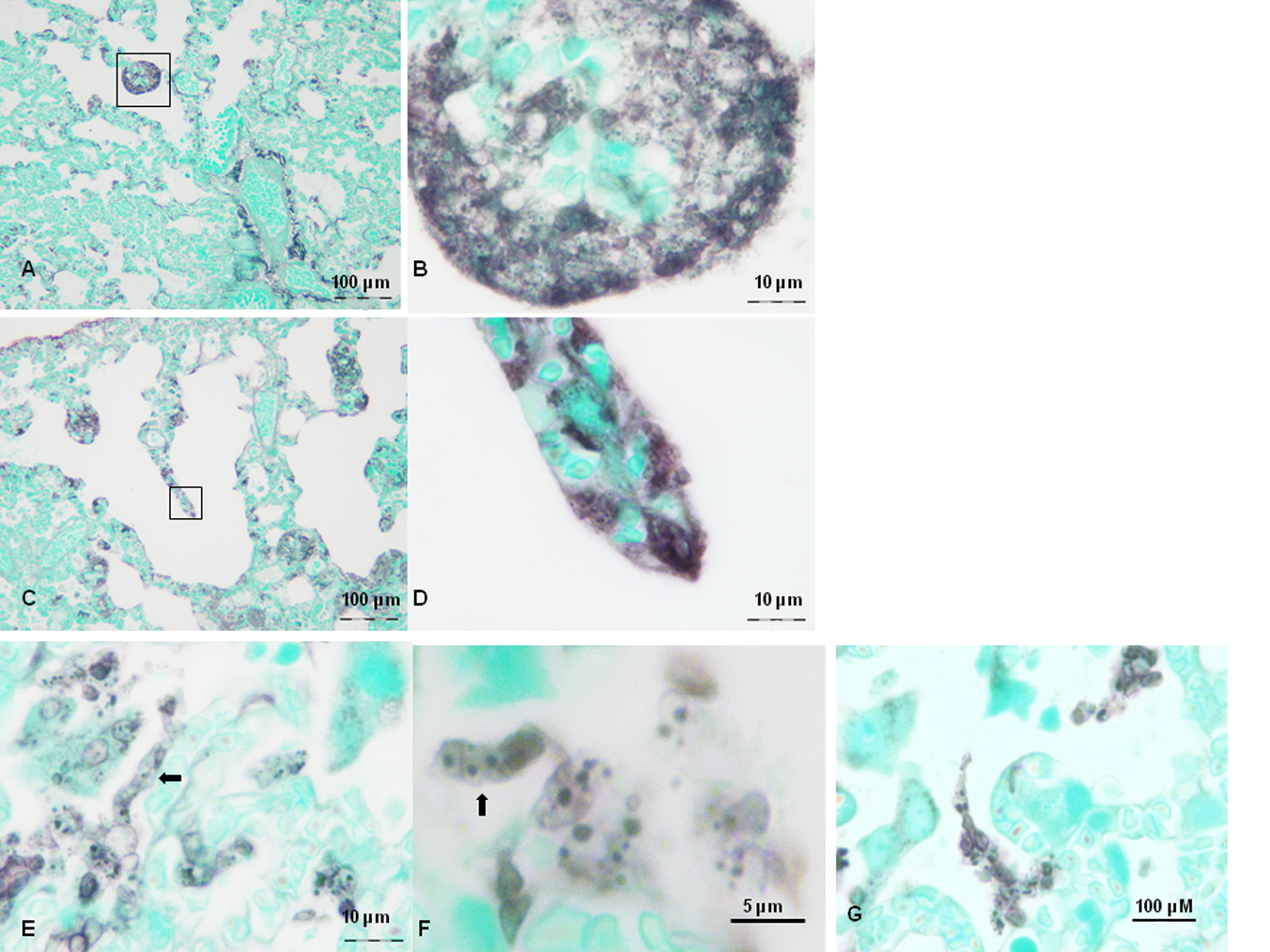

Supplement: Figure S1 — Unusual morphologies of P. murina in the lungs of mice treated with echinocandins. Grocott's methenamine silver (GMS)-stained sections of lungs from mice treated with anidulafungin and micafungin: Panel a: 10× magnification of ball-like structure in a mouse treated with 5 mg/kg anidulafungin. Note the clearly circumscribed boundaries of this ∼70 um structure. Panel b: Higher magnification of the structure. Panel c: Extension containing GMS-stained organisms in a mouse treated with 5 mg/kg micafungin. Panel d: Higher magnification of the tip of the elongation (1250X). Panel e: Chains of GMS-staining organisms resembling pseudohyphae in a mouse treated with 5 mg/kg micafungin. Panel f: Higher magnification of a different chain in the same mouse treated with 5 mg/kg micafungin. Panel g: An unusual elongated structure in a mouse treated with 5 mg/kg micafungin. Magnification bars are included for each micrograph. (2.26 MB TIF) [file pone.0008524.s001.tif]
